# *miR-27a-3p* Targets ATF3 to Reduce Calcium Deposition in Vascular Smooth Muscle Cells

**DOI:** 10.1016/j.omtn.2020.09.030

**Published:** 2020-09-28

**Authors:** Nakwon Choe, Duk-Hwa Kwon, Juhee Ryu, Sera Shin, Hye Jung Cho, Hosouk Joung, Gwang Hyeon Eom, Youngkeun Ahn, Woo Jin Park, Kwang-Il Nam, Young-Kook Kim, Hyun Kook

**Affiliations:** 1 Department of Pharmacology, Chonnam National University Medical School, Hwasun, Jeollanamdo, Republic of Korea; 2 Department of Biochemistry, Chonnam National University Medical School, Hwasun, Jeollanamdo, Republic of Korea; 3 Department of Anatomy, Chonnam National University Medical School, Hwasun, Jeollanamdo, Republic of Korea; 4 Department of Cardiology, Chonnam National University Hospital, Gwangju, Republic of Korea; 5 College of Life Sciences, Gwangju Institute of Science and Technology, Gwangju, Republic of Korea

**Keywords:** vascular calcification, vascular smooth muscle cells, miRNA, miR-27a-3p, activating transcription factor 3

## Abstract

Vascular calcification, the ectopic deposition of calcium in blood vessels, develops in association with various metabolic diseases and atherosclerosis and is an independent predictor of morbidity and mortality associated with these diseases. Herein, we report that reduction of *microRNA-27a-3p* (*miR-27a-3p*) causes an increase in activating transcription factor 3 (ATF3), a novel osteogenic transcription factor, in vascular smooth muscle cells. Both microRNA (miRNA) and mRNA microarrays were performed with rat vascular smooth muscle cells, and reciprocally regulated pairs of miRNA and mRNA were selected after bioinformatics analysis. Inorganic phosphate significantly reduced the expression of *miR-27a-3p* in A10 cells. The transcript level was also reduced in vitamin D_3_-administered mouse aortas. *miR-27a-3p mimic* reduced calcium deposition, whereas *miR-27a-3p inhibitor* increased it. The Atf3 mRNA level was upregulated in a cellular vascular calcification model, and *miR-27a-3p* reduced the *Atf3* mRNA and protein levels. Transfection with *Atf3* could recover the *miR-27a-3p*-induced reduction of calcium deposition. Our results suggest that reduction of *miR-27a-3p* may contribute to the development of vascular calcification by de-repression of ATF3.

## Graphical Abstract

**Figure:**
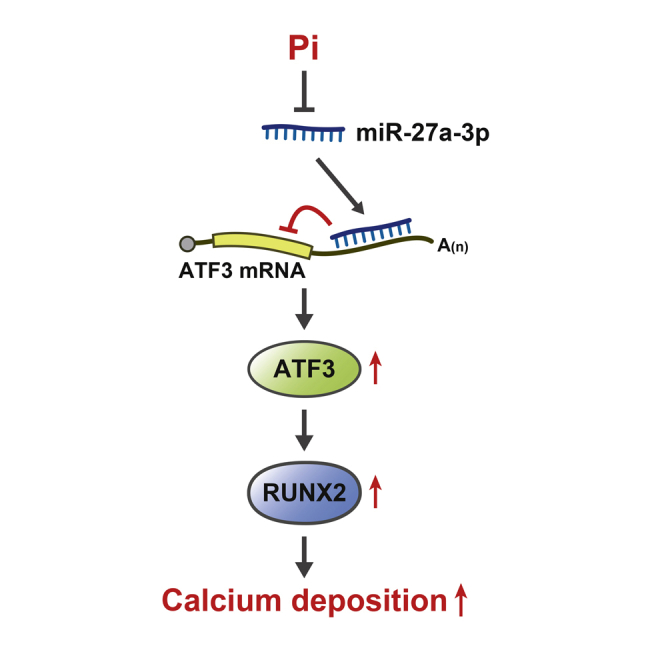

## Teaser

Vascular calcification causes stiffness of blood vessels, which causes secondary damage to the cardiovascular system. A reduction in the microRNA miR-27a-3p leads to an increase in the transcription factor ATF3, which induces calcium deposition. This miR-27a-3p/ATF signaling pathway may provide a novel therapeutic target for vascular calcification.

## Introduction

In abnormal circumstances, hydroxyapatite, a calcium phosphate mineral, can be deposited in blood vessels, which is linked to aging and cardiovascular or metabolic diseases such as atherosclerosis, chronic kidney disease, and diabetes.^1^ This phenomenon, called vascular calcification, causes remodeling of the blood vessels, resulting in an increase in stiffness. Because this abnormal rigidity itself can cause secondary fatal diseases such as ischemia of distal tissue or rupture, vascular calcification is often considered an independent risk factor for cardiovascular events.^2^

Anatomically, vascular calcification can be divided into two types, intimal calcification and medial calcification, which depend on the location of the calcification in the blood vessels. Intimal calcification is associated with atherosclerosis and thereby can cause arterial obliteration or atherosclerotic plaque rupture. Medial calcification, however, induces vascular stiffness, hypertension, and an increase in pulse pressure, which can cause diastolic dysfunction of the heart.^3^, ^4^, ^5^ While atherosclerosis is highly linked with intimal calcification, diabetes (types 1 and 2) and its associated metabolic syndrome as well as chronic renal failure are associated with both types of calcification (intimal and medial).^2^

Vascular smooth muscle cells (VSMCs) are a key regulator of vascular calcification. In the physiologic condition, VSMCs show a contractile phenotype and highly express related proteins such as smooth muscle (SM) α-actin, SM-22α, and calponin. Upon injury or stress, however, VSMCs downregulate the expression of those contractile proteins, but they increase the expression of proliferation-associated proteins, resulting in remodeling of the blood vessels, called the “synthetic phenotype.” Traditionally, it was known that VSMCs undergo the contractile or synthetic phenotype, and vascular calcification was thought to be a consequence of passive processes following VSMC death. However, recent studies have delineated that VSMCs can differentiate into diverse cellular types such as osteoblasts, chondrocytes, adipocytes, and macrophage foam cells.^2^ Thus, vascular calcification can be considered an active process of osteogenic differentiation of VSMCs, which is caused by an imbalance in osteogenic/bone absorption mechanisms in the blood vessels.^6^

MicroRNAs (miRNAs), which are found in diverse organisms, are single-stranded noncoding RNAs with 22 nt that regulate gene expression at the post-transcriptional level by binding to the 3′ untranslated region (UTR) of mRNAs.^7^ Because a perfect match between the miRNAs and the target mRNA is not required, individual miRNAs can target multiple mRNAs, and a single mRNA can be regulated by multiple miRNAs. Thus, up to 90% of genes and related cellular functions are under their control, and, indeed, miRNAs mediate various cellular functions, including proliferation, apoptosis, and differentiation.^8^

The functions of VSMCs are also precisely regulated by many miRNAs. For example, diverse miRNAs are involved in atherogenesis and phenotype switching.^9^, ^10^, ^11^, ^12^ Our laboratory also found that *miR-132*, *miR-34c*, and *miR-124* regulate atherosclerosis by targeting Lrrfip-1, stem cell factor, and S100 calcium-binding protein A4, respectively.^13^, ^14^, ^15^ Of note, among those miRNAs, the *miR-27* family has been appreciated as one of the diagnostic or prognostic markers in vascular inflammation.^16^ Indeed, *miR-27* is involved in angiogenesis, apoptosis, lipid regulation, and cytokine production, mainly in the endothelium, which contributes to the development of atherosclerosis.^17^^,^^18^ However, the roles of *miR-27* in vascular calcification are not clear.

Many research groups have reported the roles of miRNAs in vascular calcification; in most reports, miRNA microarrays or total RNA sequencing or even a literature search was used in many vascular calcification-mimicking experimental conditions for the initial screening of miRNAs. miRNAs have diverse functions as inhibitors or activators of vascular calcification. For example, the *miR-30* family has been reported to inhibit vascular calcification.^19^, ^20^, ^21^, ^22^, ^23^ *miR-125b*^24^ and *miR-135a*^24^ are also anti-calcification miRNAs. In contrast, *miR-32*,^24^ *miR-324-3p*,^25^ and *miR-486*^26^ are examples of pro-calcification miRNAs. Interestingly, some miRNAs, such as *miR-29b* (negative^27^, ^28^, ^29^ versus positive^30^^,^^31^), have been reported to have dual action depending on the experimental conditions. Bioinformatics database searching was used to search for the target mRNA of those miRNAs, and most of the target genes found were also associated with osteoblast differentiation and osteogenic transdifferentiation. Regardless of the cause, the phenomenon of calcium deposition in VSMCs is one of the key features in the development of vascular calcification. Indeed, considering the complexity and diversity of miRNA-associated regulation, further investigation of the role of miRNAs in vascular calcification is required. Thus, in the present study, we investigated vascular calcification-related miRNAs in VSMCs. We first performed both miRNA and mRNA arrays in inorganic phosphate (Pi)-treated rat VSMCs (rVSMCs) and found that *miR-27a-3p* was downregulated, while its putative target activating transcription factor 3 (ATF-3) was upregulated. We further found that *miR-27a-3p* inhibits vascular calcification by attenuating activating transcription factor 3 (ATF3).

## Results

### Screening of Vascular Calcification-Associated miRNAs and Their Possible Targets

**Figure 1:**
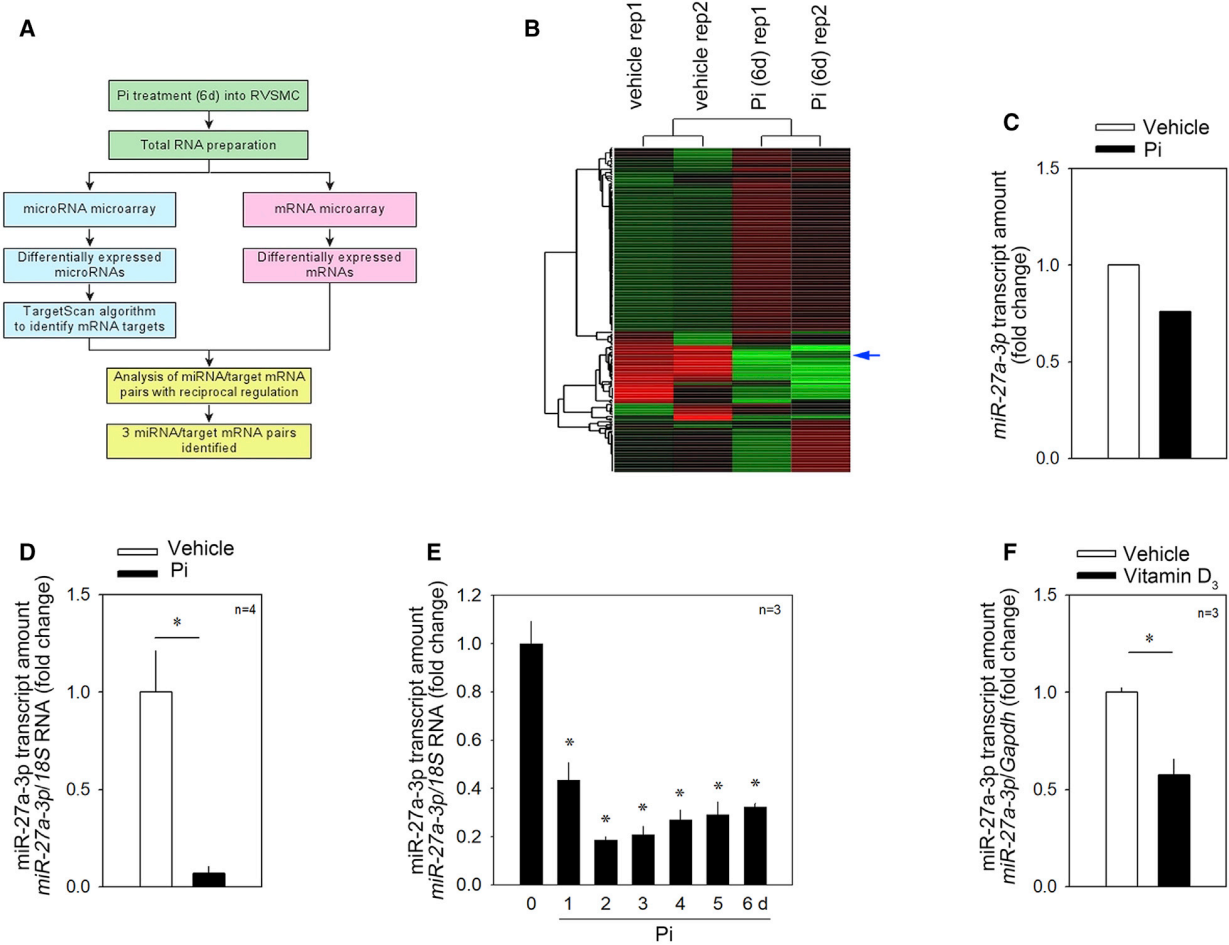
Strategies for the Discovery of Novel miRNA/Target Gene Sets and Confirmation of Changes of *miR-27a-3p* (A) Strategy to find a novel miRNA and its target. Primarily cultured rat vascular smooth muscle cells (rVSMCs) were treated for 6 days and total RNA was prepared for a miRNA microarray and mRNA microarray. The samples were duplicated for each condition. The averaged value was used for analysis. (B) Gene tree analysis with the miRNA microarray results (GEO: GSE130486). (C) Changes in *miR-27a-3p* in microarray results in rVSMCs. Two values were averaged. (D) qRT-PCR analysis. Inorganic phosphate (Pi) treatment for 6 days significantly reduced the *miR-27a-3p* transcript amount in A10 cells. (E) Time course of Pi treatment and the decrease in *miR-27a-3p* amount. (F) The *miR-27a-3p* transcript level was significantly downregulated in the aorta obtained from vitamin D_3_-administered mice. Error bars indicate SD. ∗p < 0.05, ∗∗p < 0.01.

To search for pairs of miRNAs and their targets, we performed miRNA and mRNA microarrays in Pi-treated rVSMCs. We first treated rVSMCs with Pi for 6 days and observed that Pi sufficiently induced calcium deposition (Figures S1A and S1B). We next performed a miRNA microarray (GEO: GSE130486). To find the target genes of those miRNAs, we performed a mRNA microarray, which was previously deposited (GEO: GSE74755). Among the downregulated miRNAs, we listed about eight miRNAs that might have roles in the cardiovascular system as identified by a literature search and analyzed their possible target genes by using TargetScan software. We focused on the pair of “downregulated miRNA” and “reciprocally upregulated putative miRNA-target gene” and selected three miRNAs (Figure 1A). Among them, the pair of *miR-27a-3p*, which was downregulated, and its putative target Atf3 were selected for further investigation. *miR-27a-3p* drew our interest because *miR-27a-3p* is reported to be downregulated in human calcified valves.^32^ It is also known to be associated with diverse cardiovascular diseases, such as atherosclerosis and cardiac fibrosis.^16^^,^^33^^,^^34^ However, its mechanism in association with vascular calcification has not been reported. The downregulation of *miR-27a-3p* in the miRNA microarray is shown in Figures 1B and 1C.

### Validation of Changes in *miR-27a-3p*

Next we prepared Pi-treated rVSMCs to validate the dysregulation of the miRNAs. Pi increased the calcium content of the cells 4 days after treatment, with an abrupt increase at days 5 and 6 (Figure S1C). The increase in calcium contents was accompanied with the increase in the runt-related transcription factor 2 (Runx2) mRNA level (Figure S1D). Treatment with Pi for 6 days significantly reduced the transcript level of *miR-27a-3p* (Figure 1D). The Pi-induced decrease in *miR-27a-3p* started at day 1 (Figure 1E).

We further examined whether *miR-27a-3p* is downregulated *in vivo* in a mouse model. *miR-27a-3p* was well conserved in both mice and rats. As we have previously reported,^35^ administration of vitamin D_3_ (150 μL/25 g, 5 × 10^5^ IU/kg/day) successfully induced an increase in the serum calcium level (Figure S2A) and in calcium deposition in the mouse aorta (Figure S2B). Induction of vascular calcification in the mouse model significantly reduced the expression of *miR-27a-3p* (Figure 1F).

### *miR-27a-3p* Increases VSMC Calcification

**Figure 2:**
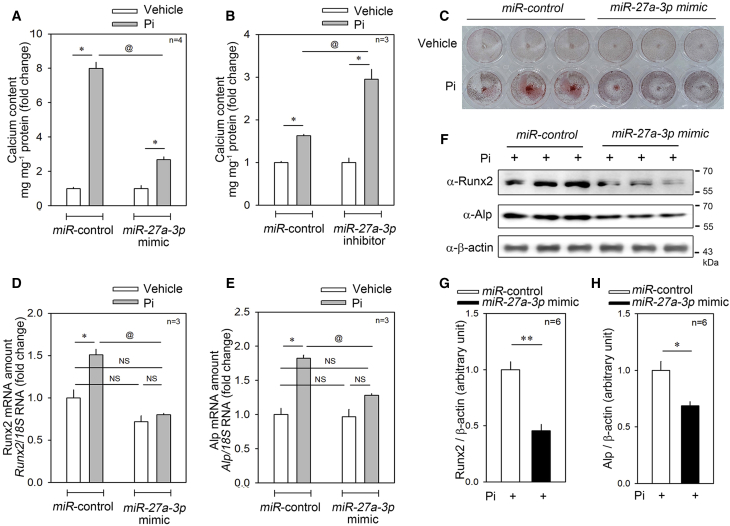
Pi-Induced Calcium Deposition Was Significantly Attenuated by Transfection with *miR-27-a-3p mimic* (A) Pi (6 days) significantly increased the calcium deposition in A10 cells, a rVSMC line. However, the increase was significantly blunted when *miR-27a-3p mimic* was transfected. (B) Pi (2 days)-induced increase in calcium deposition was potentiated by simultaneous transfection of *miR-27a-3p inhibitor*. (C) Alizarin red staining to show A10 mineralization. *miR-27a-3p mimic* blocked the Pi-induced increase in the calcium deposition. (D) The increase in the transcript level of *Runx2*, a key transcription factor of ossification, was completely abolished when *miR-27a-3p mimic* was co-transfected. (E) The increase in alkaline phosphatase (Alp) transcript level was also blocked by *miR-27a-3p mimic* transfection. (F) Western blot analysis to examine the Runx2 and Alp protein amount after transfection of *miR-27a-3p mimic*. (G and H) Quantification results of western blot analysis of Runx2 (G) and ALP (H) in the presence of Pi. Error bars indicate SD. ∗p < 0.05, ^@^p < 0.05, ∗∗p < 0.01. NS, not significant.

We next questioned the biological meaning of the reduction in the *miR-27a-3p* level in vascular calcification. To do this, we first checked whether *miR-27a-3p* affects calcification in A10 cells. When control miRNA was transfected, treatment with Pi for 6 days induced an 8-fold increase in the calcium contents in A10 cells. However, the increase was significantly blunted when *miR-27a-3p mimic* was transfected (Figure 2A). Treatment with Pi for 2 days increased the calcium content by 1.8-fold; the increase was significantly potentiated by transfection of *miR-27a-3p inhibitor* (Figure 2B). Alizarin red staining further showed that the mimic reduced the calcium content in the presence of Pi (Figure 2C).

Vascular calcification is related to osteogenic differentiation of VSMCs of mesenchymal origin.^36^ RUNX2 is a key transcription factor of osteoblast differentiation, and its ectopic expression in VSMCs is one of the key features in vascular calcification.^37^ We next checked the expression of Runx2 in A10 cells in the presence of *miR-27a-3p mimic*. First, Pi treatment significantly increased the expression of *Runx2* mRNA. However, transfection of *miR-27a-3p mimic* abolished the increase (Figure 2D). As in bone formation, in order to mineralize the extracellular matrix, vascular calcification requires the release of matrix vesicles that contain alkaline phosphatase (ALP), mainly from VSMCs.^38^ By phosphatase activity-dependent blockade of calcification inhibitors such as inhibitory polyphosphates and osteopontin, ALP induces vascular calcification. Thus, ALP is a key marker of the severity of vascular calcification in association with chronic kidney disease.^39^ Indeed, in our experimental model, Pi treatment significantly increased the mRNA expression of *Alp*. However, the increase was significantly blocked when *miR-27a-3p mimic* was transfected (Figure 2E). The changes in protein expression of Runx2 and ALP further supported that *miR-27a-3p* works to inhibit Pi-induced vascular calcification (Figure 2F). The quantitative results of those changes in Runx2 and Alp expressions are shown in Figures 2G and 2H, respectively.

### *miR-27a-3p* Targets ATF3

**Figure 3:**
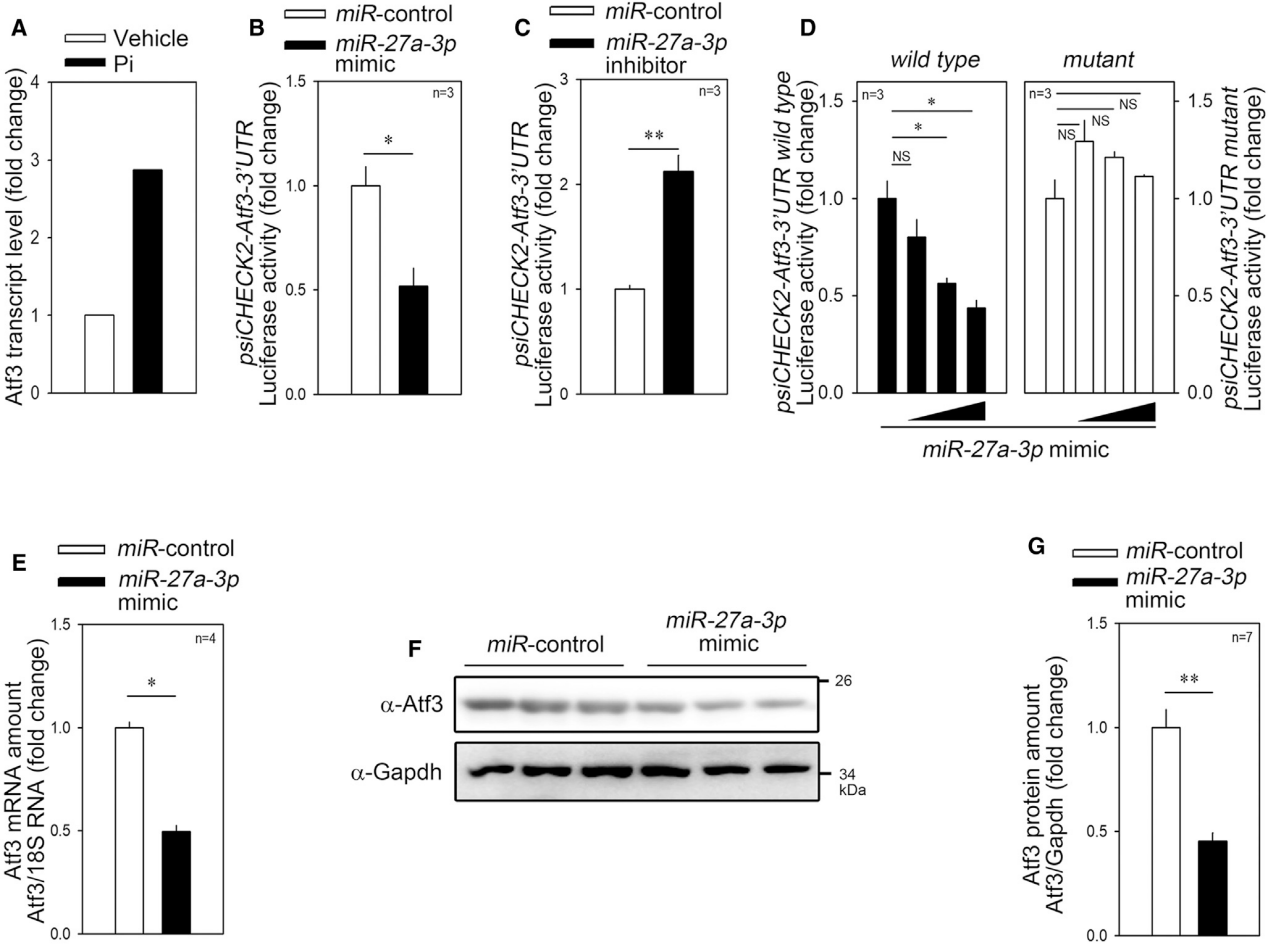
*miR-27a-3p* Targets the 3′ UTR of *ATF3* (A) Atf3 transcript level in primarily cultured rVSMCs treated with Pi. The results were obtained from an mRNA microarray (GEO: GSE74755). (B) *miR-27a-3p mimic* significantly attenuated the luciferase activity driven by *Atf3*-3′ UTR. psiCHECK2*-Atf3-*3′ UTR was used for luciferase activity measurement. (C) *miR-27a-3p inhibitor* dramatically increased the luciferase activity. (D) *miR-27a-3p mimic* failed to inhibit mutant *Atf3-*3′ UTR where the *miR-27a-3p*-binding sequence was altered. (E) *miR-27a-3p mimic* significantly reduced the mRNA transcript amount of Atf3. (F and G) *miR-27a-3p mimic* reduced the protein amount of Atf3 as determined by western blot analysis. Quantification results from the western blot (F) are shown in (G). Error bars indicate SD. ∗p < 0.05, ∗∗p < 0.01. NS, not significant.

We screened target candidates of *miR-27a-3p in silico* to identify genes related to vascular calcification. miRNAs inhibit their target genes by binding to the 3′ UTR, resulting in the degradation of target mRNA.^40^ In these experiments, among the putative target genes, we selected only those upregulated in the mRNA microarray in the same *in vitro* vascular calcification condition in which *miR-27a-3p* was downregulated (Figure 1A). ATF3, a putative target of *miR-27a-3p*, was upregulated, which met the criteria (Figure 3A).

The alignment of *miR-27a-3p* and the 3′ UTR of *ATF3* is demonstrated in Figure S3A. Next, we generated a luciferase construct by inserting the 3′ UTR of *Atf3* into the psiCHECK2 vector and co-transfected the plasmid with either *miR-27a-3p mimic* or *inhibitor* to observe whether luciferase activity was altered. The *miR-27a-3p mimic* successfully attenuated luciferase activity (Figure 3B), while the *miR-27a-3p inhibitor* increased it (Figure 3C). To check whether the *miR-27a-3p*-mediated repression of psiCHECK2-Atf3-3′ UTR luciferase activity was sequence-specific, we generated a mutant psiCHECK2-Atf3-3′ UTR by introducing a mutant sequence as shown in Figure S3B. *miR-27a-3p mimic* failed to repress the mutant luciferase construct (Figure 3D). We further investigated the effect of *miR-27a-3p mimic* on the *Atf3* mRNA level. *miR-27a-3p mimic* significantly reduced the *Atf3* mRNA level (Figure 3E). We also saw the inhibitory effect when we checked the protein amount. Transfection of *miR-27a-3p mimic* significantly attenuated the protein amount of Atf3 as determined by western blot analysis (Figure 3F). The quantification results are shown in Figure 3G.

### Pi-induced Upregulation of Atf3 Is Blocked by *miR-27a-3p*

**Figure 4:**
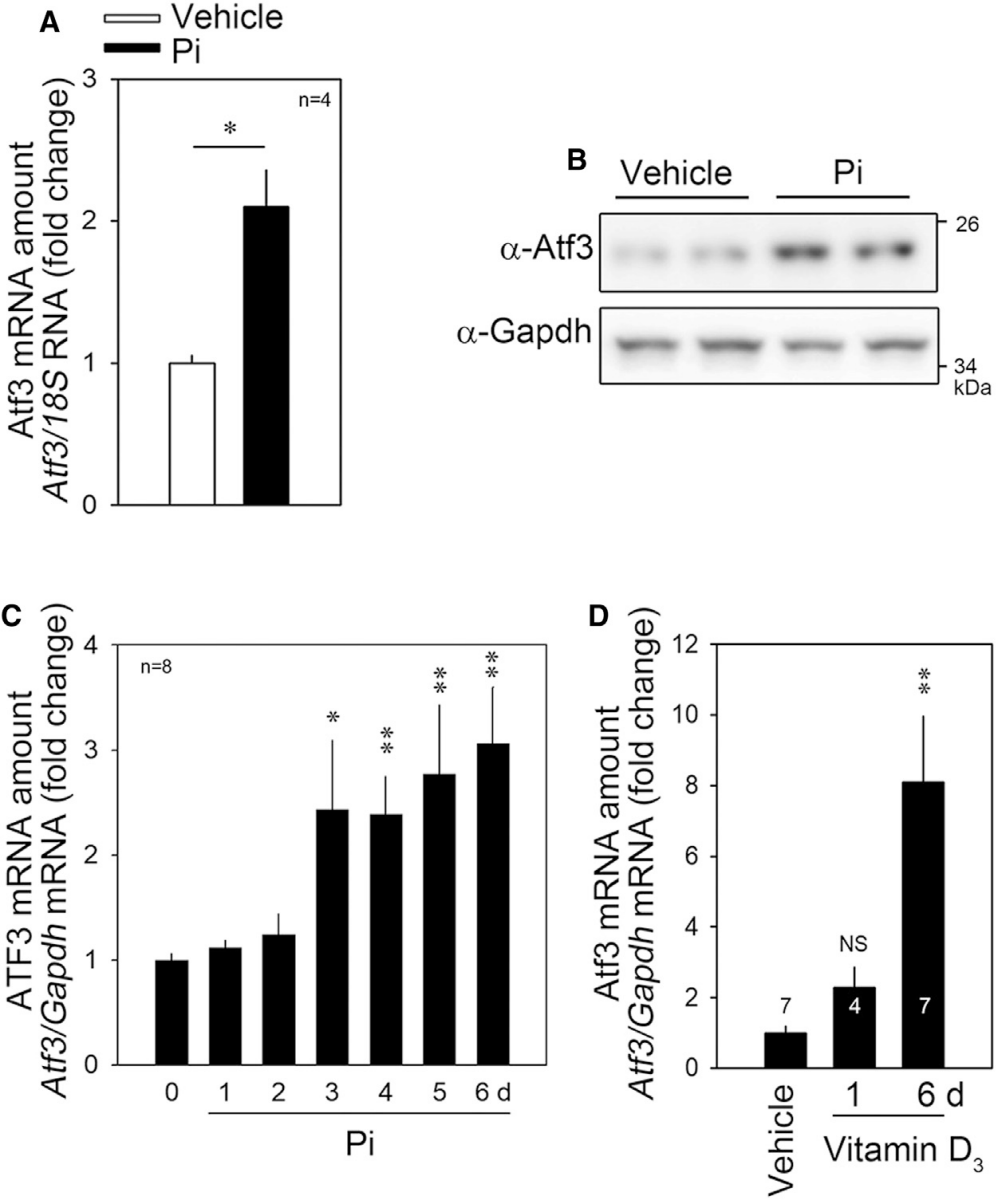
Pi Induces Atf3 Expression, Which Is Attenuated by *miR-27a-3p* (A and B) Pi (6 days) increased the Atf3 transcript amount as determined by quantitative RT-PCR (A) or by western blot analysis (B). (C) The Atf3 transcript amount was gradually increased by Pi in a time-dependent fashion. (D) Atf mRNA was also increased by vitamin D_3_ administration in mice. Numbers in bars represent the numbers of mice tested. Error bars indicate SD. ∗p < 0.05, ∗∗p < 0.01.

To confirm the results of the mRNA microarray (Figure 3A), we checked whether Pi could induce the expression of Atf3 by quantitative real-time PCR. Treatment with Pi for 6 days increased the *Atf3* mRNA level (Figure 4A) as well as its protein amount (Figure 4B). The Pi-induced increase in Atf3 mRNA was dramatic in the relatively late phase of 6 days after Pi treatment (Figure 4C). Administration of vitamin D_3_ also induced a significant increase in *Atf3* mRNA (Figure 4D).

### Vitamin D_3_ Downregulates *miR-27a-3p* but Upregulates Atf3 in a Mouse Model

**Figure 5:**
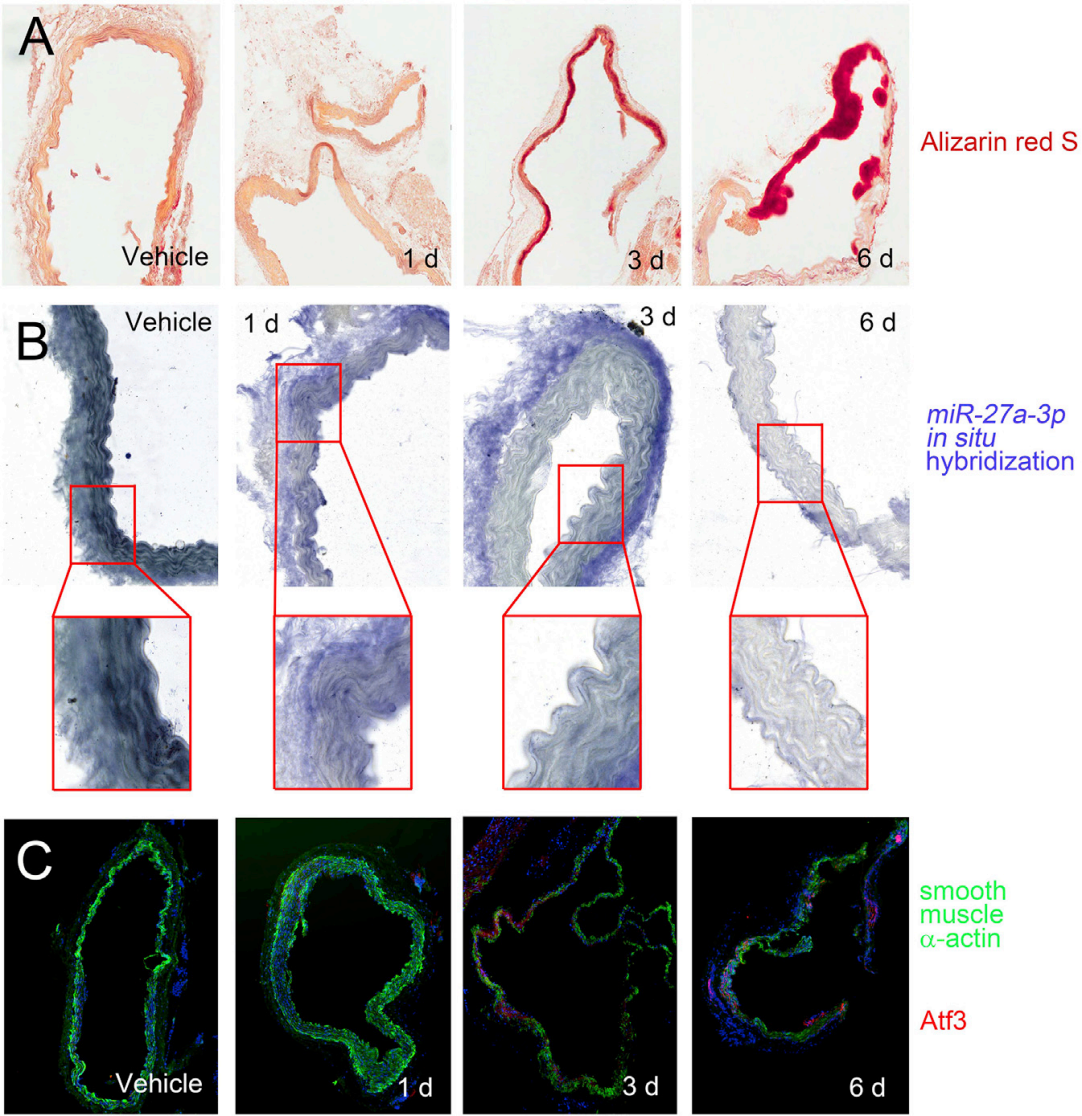
Histologic Analysis of *miR-27a-3p* and Atf3 in Vitamin D_3_-Administered Mouse Aorta (A) Alizarin red S staining shows the marked increase in calcification. (B) *In situ* hybridization analysis shows the *miR-27a-3p* transcript. Digoxin signals shown in dark blue are high in the muscle tissue as well as in the pericyte of the vehicle-administered (0 d) mice. However, the expression in the smooth muscle tissue was gradually downregulated by administration of vitamin D_3_. (C) Fluorescent immunohistochemistry analysis shows the expression of smooth muscle α-actin (green) and Atf3 (red). Note that the expression of α-smooth muscle actin was gradually decreased, whereas that of Atf3 was increased by day 6 of vitamin D_3_ administration.

We investigated whether the reciprocal regulation of *miR-27a-3p* and Atf3 also took place in an *in vivo* vascular calcification model. Administration of vitamin D_3_ significantly increased calcification, and the calcium deposition was prominent at 6 days after administration (Figure 5A). The expression of *miR-27a-3p* was examined by *in situ* hybridization (ISH). In vehicle-administered mouse aorta, *miR-27a-3p* was well localized in the muscular layer. It is noteworthy that *miR-27a-3p* is also highly expressed in vascular pericytes (Figure 5B).

SM22α is highly expressed in the contractile phenotype, whereas it is downregulated in the case of vascular calcification.^41^ In the present study, vehicle-administered mouse aorta showed high expression of SM22α (green color, 0 day, Figure 5C). However, the expression of SM22α was gradually downregulated when vitamin D_3_ was administered (2–6 days). Inversely, Atf3 expression (red color) was significantly upregulated in a spot-like localized area in the smooth muscle layer (3 and 6 days, Figure 5C).

### ATF3 Induces Vascular Calcification

**Figure 6:**
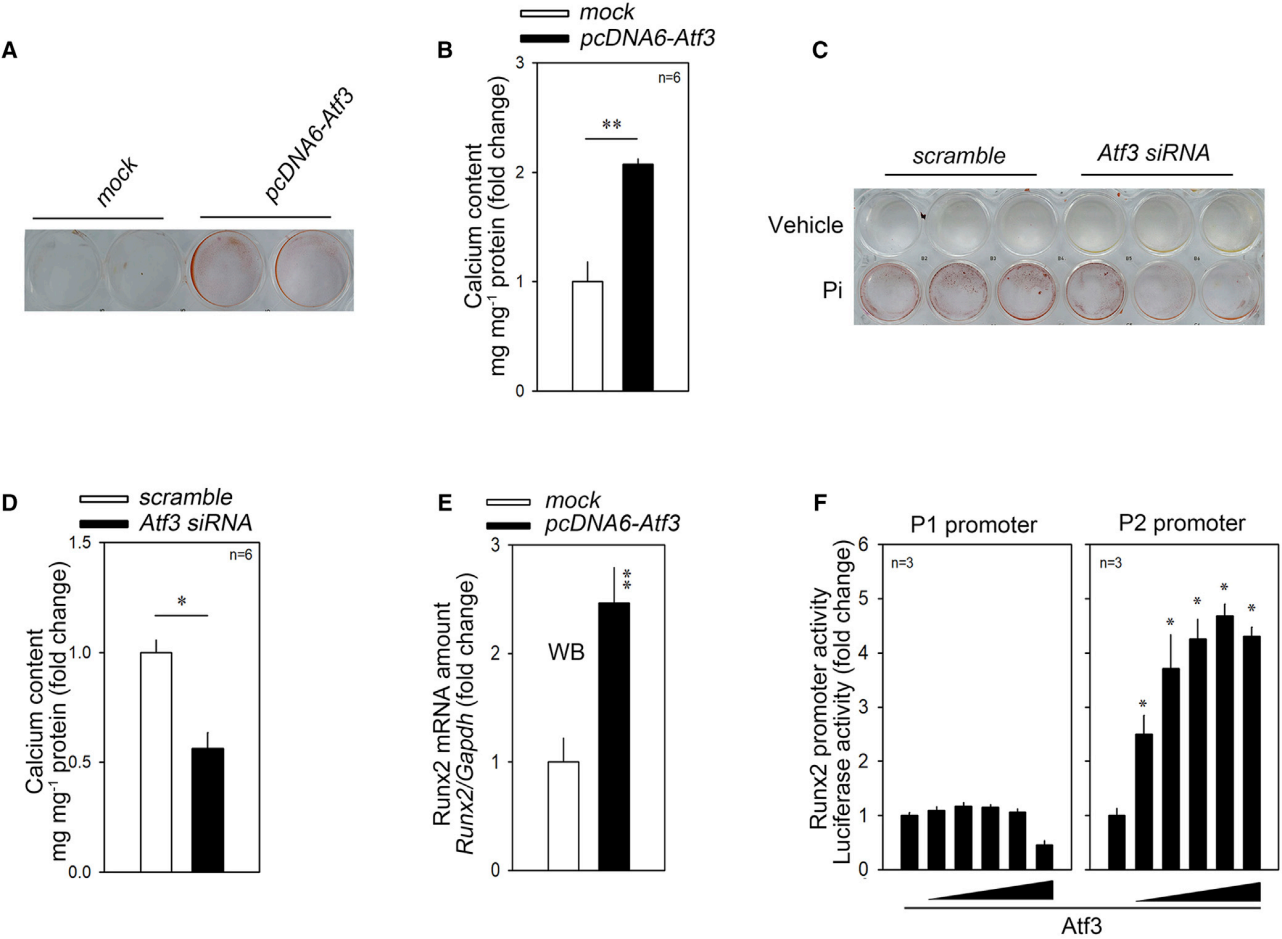
Atf3 Induces Calcium Deposition (A and B) Transfection of *Atf3* increased calcium deposition. (A) Alizarin red S staining. (B) Calcium deposition. (C and D) Atf3 siRNA significantly reduced the calcium deposition in the presence of Pi. (C) Alizarin red S staining. (D) *Atf3* siRNA significantly reduced the calcium contents in the presence of Pi. (E) Transfection of Atf3 induced an increase in the Runx2 mRNA level. (F) Promoter analysis. Atf3 increased the Runx2 P2 (proximal) promoter (right panel) in a dose-dependent fashion. Error bars indicate SD. ∗p < 0.05 ∗∗p < 0.01.

The expression of ATF3 is increased in response to serum, angiotensin II, and H_2_O_2_. The role of ATF3 in VSMCs is to prevent their apoptosis and thereby increase their survival,^42^ which implies that ATF3 may work as a compensatory mechanism against those stresses. Indeed, in our experimental model, transfection of *Atf3* increased A10 cell proliferation (Figure S4A). The involvement of ATF3 in vascular calcification, however, has not been investigated. Thus, we first checked whether ATF3 affects calcium deposition in VSMCs. Overexpression of *Atf3* significantly increased the deposition of calcium as determined by alizarin red staining (ARS) (Figure 6A) and by calcium content measurements (Figure 6B).

Interestingly, ATF3 has been reported to a negative regulator of bone formation;^43^ it inhibits osteoblast differentiation by downregulation of ALP in MC3T3-E1, a preosteoblastic cell line,^44^ which may contradict our results. Thus, we checked the effect of ATF3 in MC3T3-E1 cells. Transfection of *pcDNA6-Atf3-myc* did not alter the calcium contents; however, it significantly attenuated the Pi-induced calcium deposition (Figure S4B). These results suggest that ATF3-induced calcium deposition is cell type-specific.

We further checked whether knocking down Atf3 may inhibit calcium deposition. Small interfering RNA (siRNA)-mediated reduction of Atf (Figure S4C) dramatically reduced the calcium deposition in the presence of Pi (Figures 6C and 6D).

We next investigated whether ATF3 can induce Runx2; transfection of Atf3 significantly increased the Runx2 mRNA level (Figure 6E). We further examined whether the increase in Atf3 mRNA was caused by transactivation of the Runx2 promoter. Runx2 has two different transcripts, Runx2 type II and Runx2 type I, which are transcriptionally regulated by two distinct promoters, the distal P1 and the proximal P2 promoters, respectively.^45^ The Runx2 type II transcript is exclusively expressed in bone precursor cells, whereas Runx2 type I is expressed in both osteogenic and nonosteogenic cells. Promoter luciferase analysis showed that transfection of *Atf3* increased the *Runx2* P2 promoter activity in a dose-dependent fashion, whereas it failed to do so for the P1 promoter (Figure 6F). These results suggest that Atf3 upregulates calcium deposition by transcriptional activation of Runx2.

### *miR-27a-3p*-Induced Reduction in Calcium Deposition Is Recovered by Atf3

**Figure 7:**
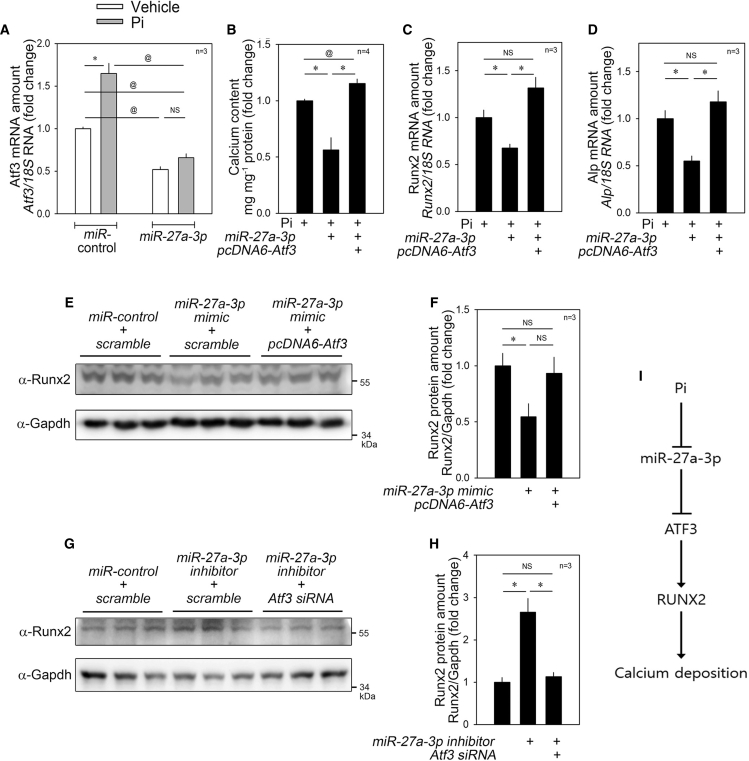
Anti-Calcification Effect of *miR-27a-3p* Is Blocked by Atf3 (A) Effect of *miR-27a-3p mimic* on *Atf3* transcript amount. The Pi-induced increase as well as the basal level of *Atf3* mRNA were significantly attenuated by transfection of *miR-27a-3p mimic*. (B) *miR-27a-3p mimic*-induced reduction of calcium deposition in the presence of Pi was completely blocked when Atf3 was overexpressed. (C and D) Changes in mRNA levels of *Runx2* (C) and *Alp* (D). (E and F) *miR-27a-3p mimic*-induced reduction of Runx2 protein amount was recovered by co-transfection of *pcDNA6-Atf3-myc*. Quantitative results of (E) was shown in (F). (G and H) *miR-27a-3p inhibitor*-induced increase in Runx2 protein amount was blocked by simultaneous transfection of *Atf3* siRNA. Quantitative results of (G) are shown in (H). (I) Diagram. Pi reduces the expression of *miR-27a-3p*, which results in the activation of ATF3, a novel pro-calcific mediator. Error bars indicate SD. ∗p < 0.05, ∗∗p < 0.01, ^@@^p < 0.01. NS, not significant.

To test whether the effect of Pi on the increase in Atf3 (Figure 4A) is *miR-27a-3p*-dependent, we transfected *miR-27a-3p* and observed that the Pi-induced increase in Atf3 mRNA was blocked (Figure 7A), which suggested that Atf3 expression could be regulated by *miR-27a-3p*. In addition, in order to examine whether the *miR-27a-3p*-induced reduction in calcium deposition (Figure 2) was related to Atf3, we simultaneously transfected both *miR-27a-3p mimic* and *pcDNA6-Atf3-myc*. The miRNA-induced reduction in calcium deposition was fully recovered when Atf3 was overexpressed (Figure 7B). Likewise, the reduction of mRNA amounts of *Runx2* (Figure 7C) and *Alp* (Figure 7D) were completely blocked when *pcDNA6-Atf3-myc* was co-transfected.

We further checked the protein amounts of Runx2. *miR-27a-3p mimic*-induced reduction of Runx2 was attenuated (Figure 7E). Quantification results of the western blot analysis showed that transfection of *pcDNA6-Atf3-myc* restored the Runx2 protein amount (Figure 7F). We also checked the changes in the level of Runx2, a key transcription factor; the *miR-27a-3p inhibitor*-induced increase in the protein levels of Runx2 was blunted by co-transfection of *Atf3* siRNA (Figures 7G and 7H). These results indicate that *miR-27a-3p* targets ATF3 to inhibit calcium deposition in VSMCs.

One may question whether *miR-27a-3p* directly binds to the 3′ UTRs of Runx2 and Alp and thereby reduces the mRNA stability to reduce the mRNA and protein amounts of those target genes. To rule out these possibilities, we generated psiCHECK2-Runx2-3′ UTR luciferase and psiCHECH2-Alp2-3′ UTR *luciferase* constructs and examined whether *miR-27a-3p* reduced the luciferase activity as it did for Atf3 (as shown in Figure 6G). *miR-27a-3p mimic* did not reduce their luciferase activities (Figures S5A and S5B). These results are somewhat predictable, since no clear putative *miR-27a-3p*-binding sequences were found in those 3′ UTRs (http://www.targetscan.org/vert_72/).

## Discussion

The present study suggests a novel mechanism for *miR-27a-3p* and its target Atf3 in the regulation of vascular calcification. The main finding of this work is that Pi, an inducer of osteogenic transformation of VSMCs, causes a reduction in *miR-27a-3p* and the succeeding upregulation of Atf3. We also observed that Atf3 causes calcium deposition in VSMCs (Figure 7I). Some miRNAs have been identified to be altered upon vascular calcification stresses in animal models or human diseases. For example, chronic downregulation of *miR-181b* in association with aging is known to activate transforming growth factor β (TGF-β) in VSMCs, which results in phenotypic changes. *miR-181b* directly targets TGF-β to destabilize its mRNA.^46^ In contrast, *miR-203* is upregulated by aging, which results in stiffness of blood vessels by targeting extracellular signal-regulated kinase (ERK) signaling.^47^ Indeed, several miRNAs are known to affect arterial calcification. Notably, transcription factors such as RUNX2, MSX2, KLF4, and STAT3 could be a direct target of those miRNAs that either increase or decrease vascular calcification.^21^^,^^48^ Herein, we are the first to report that *miR-27a-3p* negatively affects calcium deposition.

*miRNA-27* has two members, *miR-27a*, an intergenic miRNA, and *miR-27b*, an intronic miRNA. Twenty nucleotides out of 21 are identical in those isoforms (http://microrna.sanger.ac.uk/). As with many other miRNAs, *miR-27* is primarily involved in tumor development by affecting cell cycle or survival regulators. Interestingly, the serum level of *miR-27* is closely related to cardiovascular diseases in patients, which suggests its possible application as a biomarker in disease.^49^ In addition, *miR-27* is highly associated with atherosclerosis and its processes such as lipid metabolism, oxidative stress, inflammation, angiogenesis, shear stress, adipogenesis, and insulin resistance.^16^^,^^42^ For its anti-atherosclerotic mechanism, however, *miR-27* seems to mainly affect vascular endothelial cells, macrophages, or adipocytes, rather than VSMCs.

In this study, we identified ATF3 as a target of *miR-27a-3p* in the response of vascular calcification stress. ATF3 is a transcription factor associated with adaptive response and belongs to the ATF/cyclic AMP (cAMP) response element binding protein (CREB) family of transcription factors.^50^ ATF3 acts as either an activator or a repressor of transcription.^51^ Although its expression level is low in quiescent states, ATF3 expression is induced in response to stress conditions such as DNA damage, stimulation with cytokines or chemokines, or exposure to toxins, hypoxia, or ischemia/reperfusion injury.^50^, ^51^, ^52^ The roles of ATF3 in the cardiovascular system, however, are somewhat controversial; for example, ablation of ATF3 has been shown to exaggerate cardiac hypertrophy via ERK/c-Jun N-terminal kinase (JNK)^53^ or Egr1,^54^ whereas its overexpression also induces cardiac hypertrophy and arrhythmia.^55^^,^^56^ This discrepancy may result either from a dual role of ATF in transcription^57^ or from the contribution of other cellular types in the disease models. Indeed, macrophages are involved in cardiac hypertrophy even in the mice with cardiomyocyte-specific overexpression of ATF3.^58^

In contrast to osteogenic ATF4,^59^ ATF3 is known to block bone formation either by preventing osteoblast differentiation^43^ or by activating osteoclast activity.^60^ We also obtained the same results that ATF3 inhibits calcium deposition in MC3T3-E1 cells in our experimental models (Figure S4B). Considering that ectopic ossification in VSMCs shares many common pathways with bone formation, which is mainly mediated by activation of osteoblasts, our results showing that ATF3 induces vascular calcification (Figure 6) somewhat contradict this osteogenesis-preventing property of ATF3. However, the bidirectional transcription activity of ATF3 or cell type-specific effects may contribute to the discrepancies in VSMCs and osteoblasts in the aspect of calcium deposition. These opposite responses in either VSMCs or osteoblasts can be exemplified with estrogen-related receptor γ (ERRγ). ERRγ induces calcium deposition by upregulation of BMP2 expression in vascular smooth muscles,^61^ whereas it prevents bone formation.^62^

As ATF3 prevents cardiomyocyte apoptosis,^63^ it has been reported that ATF3 inhibits apoptosis by regulation of cytochrome *c* release.^42^ In an atherosclerosis model, ATF3 also exaggerates neointimal proliferation by enhancing VSMC proliferation.^64^ We also found that ATF3 induced A10 cell proliferation (Figure S4A). Thus, it is not likely that VSMC apoptosis and the resultant passive ectopic ossification is involved in our ATF3-induced vascular calcification. Rather, ATF3 may actively participate in the development of vascular calcification. It is noteworthy that active release of matrix metalloproteinases (MMPs) is an important step for calcium deposition.^65^^,^^66^ Although the involvement of ATF3 in vascular calcification has not been previously reported, considering that ATF3 induces VSMC migration by activating MMPs,^42^ the activation of those MMPs might also contribute to the calcium deposition in our experimental models. However, which isotypes of MMPs are involved in the ATF3-regulated release should be determined. In addition, ATF3-mediated upregulation of RUNX2 may lead us to consider the indirect transcriptional activation of those MMPs.

In the current study, we suggest that a miRNA is an upstream regulator of ATF3. Indeed, an alternate miRNA, *miR-342-3p*, targets ATF3 to promote osteogenic differentiation of human mesenchymal stem cells,^67^ which implicates the existence of a diverse miRNA-ATF3 network. In this study, we did not address which upstream signals affect the decrease in the expression of *miR-27a-3p*. Together with pathologic stresses to induce ATF3, however, *miR-27a-3p* could be one of the regulators of ATF3 expression. Alternatively, *miR-27a-3p* may intervene as a mediator between exogenous stresses and ATF3.

## Materials and Methods

All experimental procedures were approved by the Chonnam National University Medical School Research Institutional Animal Care and Use Committee and followed the National Institutes of Health *Guide for the Care and Use of Laboratory Animals* (NIH publication no. 8023, revised 1978).

### miRNA Mimic, Inhibitor, siRNA, and Antibodies

*miR-27a-3p mimic* and *inhibitor*, control miRNA, *Atf3* siRNA, and control siRNA were purchased from Bioneer (Daejeon, Korea). Antibodies against Atf3 (sc-81189, Santa Cruz Biotechnology, CA, USA), Runx2 (23981, Abcam, Cambridge, UK), Alp (108337, Abcam), β-actin (47673, Cell Signaling Technology, Danvers, MA, USA), and glyceraldehyde 3-phosphate dehydrogenase (GAPDH) (G9545, Sigma, St. Louis, MO, USA) were used at 1:1,000 dilution.

### miRNA and mRNA Microarray and Bioinformatics

To investigate changes in levels of miRNA in Pi-treated rVSMCs, an miRNA microarray was performed after pooling of three samples. RNA was isolated as described below. To reduce the experimental error, two pooled samples were independently used for the miRNA microarray and the averaged values were evaluated further. The samples were analyzed utilizing an Agilent Technologies custom rat miRNA microarray (Agilent-050340) and the results were deposited in the Gene Expression Omnibus (GEO) database under accession code GEO: GSE130486.

mRNAs that could be miRNA targets were tested by utilizing the mRNA microarray (Agilent-028282) and the results were previously deposited in the GEO database under accession code GEO: GSE74755.^35^ Putative target mRNAs of miR-27a were screened using software (http://www.targetscan.org/, http://mirtarbase.cuhk.edu.cn/php/search.php, and http://mirdb.org/).

For the clustering analysis of miRNA microarray data, Cluster 3.0 was used for the unsupervised hierarchical clustering.^68^ The analysis result was visualized by Java TreeView.^69^ In the clustering analysis, microarray signals were median-centered and normalized for genes and arrays. Average linkage analysis was performed using the centered-correlation method.

### Cloning

The coding sequence of rat Atf3 was cloned onto the pcDNA6/myc-His vector (V22120, Thermo Fisher Scientific) for overexpression of Atf3 in mammalian cells. The DNA fragment corresponding to the 3′ UTR of Atf3 mRNA containing the putative binding site for *miR-27a-3p* was cloned into the psiCHECK2 luciferase vector (C801A, Promega, Madison, WI, USA) for the luciferase assay (CosmoGeneTech, Seoul, Korea). To check whether *miR-27a-3p* affects Runx2 and Alp mRNA stability, both the 3′ UTR of *Runx2* mRNA and the 3′ UTR of *Alp* mRNA were cloned into the psiCHECK2 luciferase vector.

### Cell Cultures

rVSMCs were isolated from the thoracic aorta of 6- to 7-week-old male Sprague-Dawley rats euthanized by anesthesia with 2,2,2-tribromoethanol (240 mg/kg; intraperitoneal [i.p.] injection) (T48402, Sigma, St. Louis, MO, USA). The aorta was washed using ice-cold phosphate-buffered saline (PBS) before incubation in Ham’s F12 medium (12-615F, Lonza, Alpharetta, GA, USA) with 0.2% collagenase I (LS004196, Worthington, Lakewood, NJ, USA) at 37°C for 30 min. The aorta was opened longitudinally and the intima was scraped from the luminal surface. Tissue samples were minced in Ham’s F12 media containing 300 U/mL penicillin and 300 U/mL streptomycin and then incubated in 0.2% collagenase I solution at 37°C for 30 min. The rVSMCs were cultured in DMEM (LM001-05, Welgene, Gyeongsan, Korea) with 10% fetal bovine serum (FBS) (S001-07, Welgene) and antibiotics (15240062, Thermo Fisher Scientific, Waltham, MA, USA). rVSMCs were used at passages 2–6.

A10 cells, derived from embryonic rat aorta, were purchased from American Type Culture Collection (CRL-1476, Manassas, VA, USA) and have been used as a model system of rVSMCs. The A10 cells were cultured in DMEM with 10% FBS. All cells were maintained in an incubator under a humidified atmosphere with 5% CO_2_ at 37°C.

### Induction of Vascular Calcification *In Vitro*

The cell culture medium supplemented with 2 mM Pi was changed every 2 days for up to 6 days to induce calcification. The cells were washed twice with PBS before quantification of calcium deposition.

### Quantification of Calcium Deposition

Cells were decalcified in 0.6 N HCl at 4°C for 24 h. The calcium content of the HCl supernatants was determined using a QuantiChrom calcium assay kit (DICA-500, BioAssay Systems, Hayward, CA, USA) according to the manufacturer’s protocol. Briefly, the samples had been mixed with working reagent, and then absorbance of the mixture at 570 nm was measured using an ELx808 absorbance reader (BTELX808, BioTek Instruments, Winooski, VT, USA). Decalcified cells were lysed with 0.1 N NaOH/0.1% SDS to extract proteins. The protein content was quantified with a BCA protein assay kit (23225, Thermo Scientific/Pierce). The calcium content was normalized against the protein content.

### Observation of Calcium Deposition by Alizarin Red Staining

Cells were seeded in a 24-well plate. After treatment with Pi, the cells were washed with PBS and then fixed with 10% formalin at room temperature for 1 h. Formalin was removed by washing the cells with PBS three times. The sample was air-dried before being treated with 40 mM ARS solution (TMS-008, Sigma) for 20 min. The ARS solution was washed with distilled water and PBS until the difference between sample groups became apparent.

### Quantitative Real-Time PCR

Total RNA was extracted using either TRIzol reagent (15596026, Invitrogen, Waltham, MA, USA) or NucleoSpin RNA/protein (740933.250, Macherey-Nagel, Düren, Germany) following the manufacturer’s protocols. mRNAs and miRNAs were reverse transcribed using either a SuperScript first-strand synthesis system for RT-PCR (11904018 Invitrogen) or a TaqMan advanced miRNA cDNA synthesis kit (A28007, Applied Biosystems, Waltham, MA, USA), respectively, and then analyzed by quantitative real-time PCR using a QuantiTect SYBR Green PCR kit (204141, QIAGEN, Hilden, Germany) and a Rotor-Gene Q real-time PCR cycler (9001550, QIAGEN). *Gapdh* or *18S RNA* was used as an expression control.

### Western Blot Analysis

Cellular proteins were prepared with lysis buffer (50 mM Tris [pH 8.0], 150 mM NaCl, 1 mM EDTA, 1% Nonidet P-40 [NP-40] [28324, Thermo Fisher Scientific], 1 mM dithiothreitol [DTT], 1 mM phenylmethylsulfonyl fluoride, 1 mM Na_3_PO_4_, and protease inhibitor [11 697 498 001, Hoffmann-La Roche, Basel, Switzerland]). The proteins were separated by sodium dodecyl sulfate-polyacrylamide gel electrophoresis (SDS-PAGE) and then transferred to a polyvinylidene fluoride membrane (Millipore, Bedford, MA, USA). After blocking with 5% skim milk (232100, BD Difco, BD Biosciences, Franklin Lakes, NJ, USA) in Tris-buffered saline with Tween 20 (TBST) (20605, Thermo Scientific Fisher), the membranes were incubated with primary antibodies overnight at 4°C on a rocker. After three washes in TBST, membranes were incubated with horseradish peroxidase-conjugated secondary antibodies (7076, 7074, Cell Signaling Technology) for 1 h at room temperature. The peroxidase activity was visualized by enhanced chemiluminescence using western blotting luminol reagent (sc-2048, Santa Cruz Biotechnology) and a Fujifilm luminescent image analyzer LAS-3000 (Fujifilm Life Sciences, Richmond, VA, USA). Quantification of western blot analysis was performed after retrieving the density of the bands using Scion Image software (Scion, Frederick, MD, USA) after more than three independent sets of experiments.

### Luciferase Assay

The luciferase vector was co-transfected with *miR-27a-3p mimic* into A10 cells, and luciferase activity was measured by using a luciferase assay system (E1500, Promega) following the manufacturer’s protocols.

### *In Vivo* Vascular Calcification

Vascular calcification was induced in mice as described previously.^35^ Briefly, vitamin D_3_ (14.575 mg, 5 × 10^5^ IU/kg/day) in 70 mL of absolute ethanol was mixed with 500 mL of Cremophor (Alkamuls EL-620, Sigma-Aldrich) for 15 min. The solution was then mixed with 6.2 mL of sterilized water containing 250 mg of dextrose for 15 min. Six- to 7-week-old C57BL/6 male mice were injected with a dose of vitamin D_3_ (150 μL/25 g, 5 × 10^5^ IU/kg/day) subcutaneously for 3 days. Total RNA was extracted from the aorta of mice using TRIzol reagent following the manufacturer’s protocol for analysis.

### *In Situ* Hybridization

The isolated aortas from vitamin D_3_-treated mice, which had been euthanized with 2,2,2-tribromoethanol (240 mg/kg; i.p. injection) (T48402, Sigma, St. Louis, MO, USA), were fixed in buffered formalin at room temperature for 24 h and then embedded in paraffin. The sections (5 μm) of the fixed sample were analyzed by immunohistochemistry and *in situ* hybridization. The expression levels of *miR-27a-3p* in the arterial sections were visualized by *in situ* hybridization with an anti-*miR-27a-3p* probe (YD00614838, QIAGEN) and a miRCURY locked nucleic acid (LNA) miRNA *in situ* hybridization buffer set (339450, QIAGEN) following the manufacturer’s protocol.

Briefly, sample slides were deparaffinized with xylene and ethanol and then the sample was treated with proteinase K at 37°C for 10 min. Slides were incubated at 60°C in pre-hybridization solution for 3 h. A hybridization mixture was prepared by mixing digoxigenin (DIG)-labeled anti-miR-27a-3p probe and hybridization solution. After hybridization at 60°C overnight, the slides were washed with saline sodium citrate buffer and then incubated in blocking solution at room temperature for 1 h. Finally, ALP-conjugated anti-DIG antibody was used to detect the probe by hybridization at 4°C overnight followed by treating the slide with nitro blue tetrazolium (NBT)/5-bromo-4-chloro-3-indolyl phosphate (BCIP) at room temperature for about 4 h in a darkroom. The microscopic images were captured by a scanner (Axio Scan.Z1, Carl Zeiss, Jena, Germany).

### Immunohistochemistry

Arterial sections from the vitamin D_3_-treated mice were analyzed for protein expression by fluorescent immunohistochemistry with anti-ATF3 antibody (ab254268, Abcam) and anti-α-smooth muscle actin antibody (sc-53015, Santa Cruz Biotechnology) as described previously.^15^ The image was acquired using a Leica DM3000 microscope with a Nikon DS-Ri2 camera and Nikon NIS-Elements AR software (Nikon Instruments Korea, Seoul, Korea).

### Statistical Analysis

Data are presented as mean ± SEM. Statistical significance was determined by a Student’s t tests or one-way ANOVA, followed by a Tukey’s honestly significant difference multiple comparison *post hoc* test using PASW Statistics 19 software (SPSS, IBM, Chicago, IL, USA).

## Author Contributions

N.C., D.-H.K., J.R., S.S., and H.J. performed experiments and analyzed data. G.H.E., Y.A., and W.J.P. provided animal and cellular models and interpreted the results of experiments. H.J.C. and K.-I.N. performed *in situ* hybridization and interpreted the results. Y.-K.K. and H.K. designed the experiments and provided funding. N.C., Y.-K.K., and H.K. wrote the manuscript.

## Conflicts of Interest

The authors declare no competing interests.

## Supplemental Information

Document S1. Figures S1–S5

Document S2. Article plus Supplemental Information
