# Supplementary material for: miR-27a-3p Targets ATF3 to Reduce Calcium Deposition in Vascular Smooth Muscle Cells
Source: Mol Ther Nucleic Acids. 2020 Sep 28;22:627–39. doi: 10.1016/j.omtn.2020.09.030 (PMC7578555; doi:10.1016/j.omtn.2020.09.030)
Supplement: Document S1. Figures S1–S5 [file mmc1.pdf]

OMTN, Volume *22*

Supplemental Information

miR-27a-3p Targets ATF3 to Reduce Calcium Deposition in Vascular Smooth Muscle Cells

Nakwon Choe, Duk-Hwa Kwon, Juhee Ryu, Sera Shin, Hye Jung Cho, Hosouk Joung, Gwang Hyeon Eom, Youngkeun Ahn, Woo Jin Park, Kwang-Il Nam, Young-Kook Kim, and Hyun Kook

Figure S1

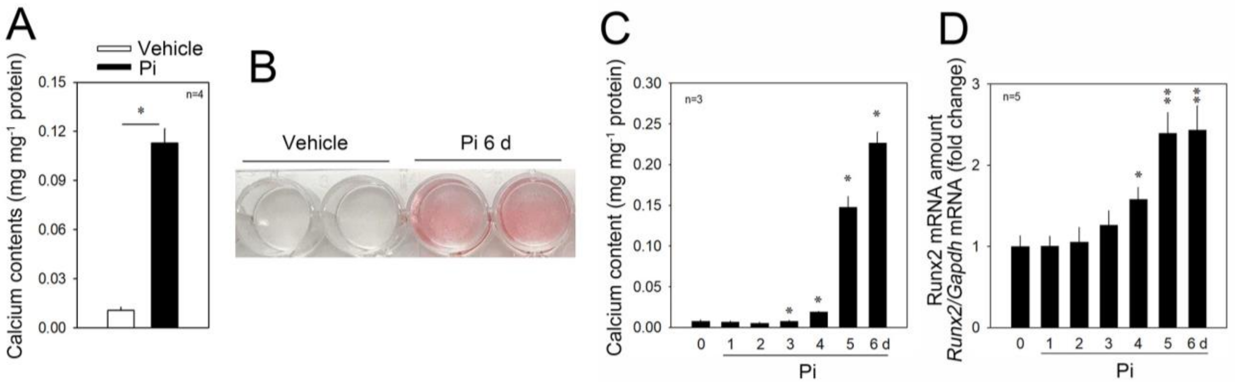

Figure S1. Inorganic phosphate (Pi) induce vascular calcification in vitro condition. (A) Treatment with inorganic phosphate (Pi) successfully induced calcium deposition in rat vascular smooth muscle cells (RVSMCs). (B) Treatment with Pi increased in a time-dependent fashion in A10 cells. (C) Alizarin red S staining shows that Pi for 6 days increased the calcium deposition. Pi: inorganic phosphate. Error bars indicate SD. * $p<0.05$, ** $p<0.01$.

Figure S2

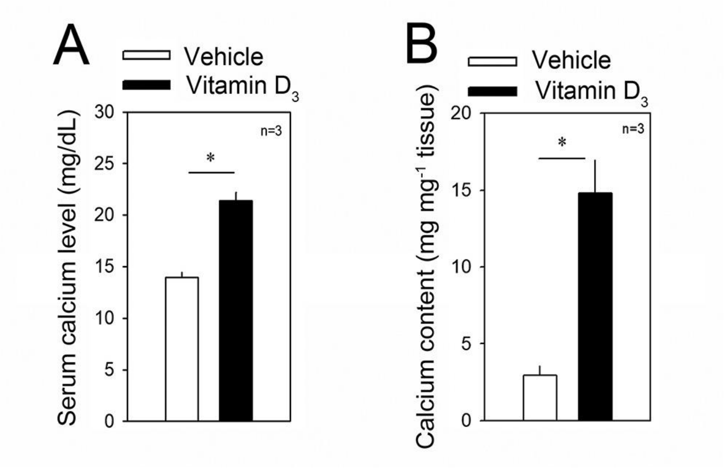

Figure S2. Vitamin D_3 induces vascular calcification in mouse aorta. Vitamin D_3 was administered daily for the first 3 consecutive days and then maintained for the following 6 days. (A) The serum calcium level was increased by vitamin D_3. (B) Calcium deposition was increased in the vitamin D_3-administered mouse aorta. Error bars indicate SD. * p<0.05, ** p<0.01.

Figure S3

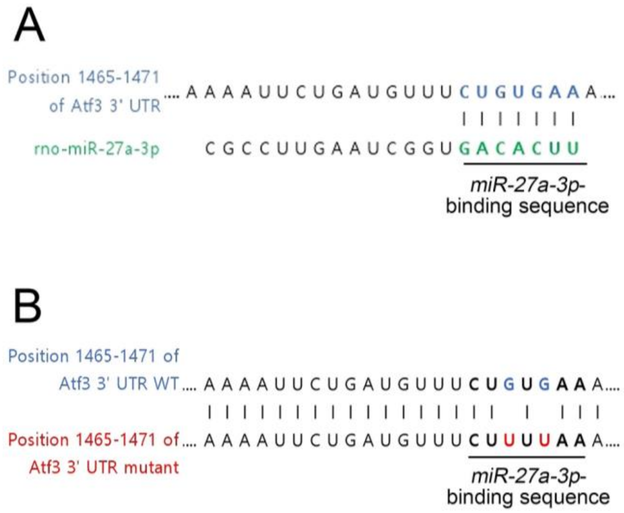

Figure S3. Sequence alignment. (A) Sequence alignments of 1465-1471 region of *Atf3* 3'UTR and *miR-27a-3p*. miR-27a-3p-binding sequence at Atf3 3'UTR was marked. (B) Sequence alignments of *psiCHECK2-Atf3 3'UTR* wild type (WT) and *psiCHECK2-Atf3 3'UTR* mutant.

Figure S4

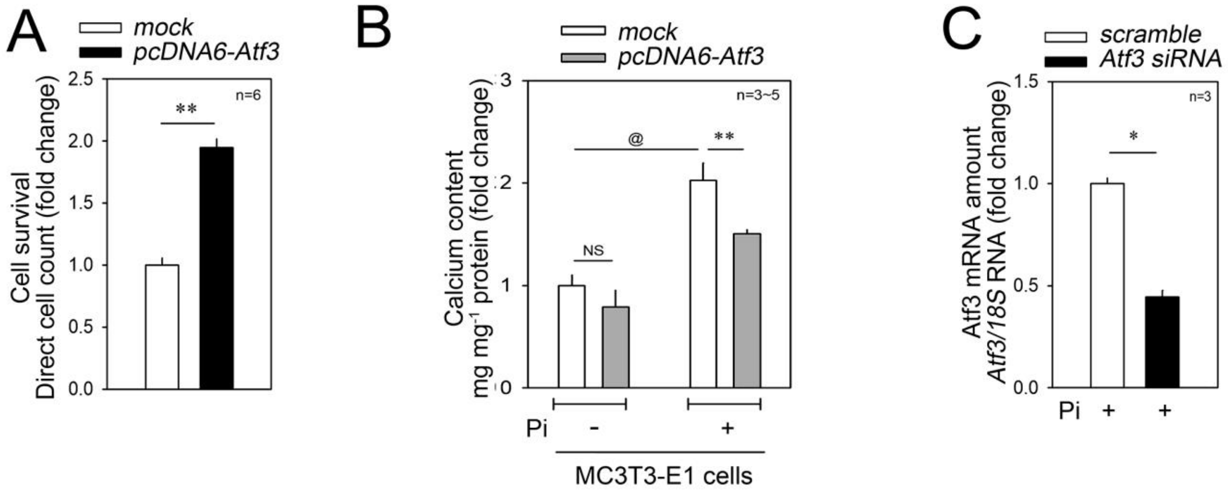

Figure S4. Effect of Atf3. (A) Transfection of *pcDNA6-Atf3-myc* increased the cell survival. (B) Atf3 blocked the Pi-induced increase in calcium deposition in MC3T3-E1 osteogenic precursor cells. (C) *Atf3 siRNA* reduced the mRNA level of Atf3 in A10 cells. Error bars indicate SD. NS: not significant, * p<0.05, ** and @@ p<0.01.

Figure S5

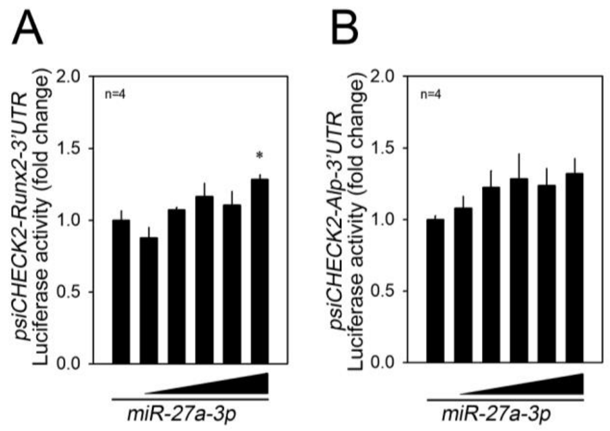

Figure S5. Effect of *miR-27a-3p mimic* transfection on luciferase activities of 3'UTRs of both Runx2 and Alp. (A) *miR-27a-3p mimic* failed to reduce *psiCHECK2-Runx2*-3'UTR luciferase activity. (B) *miR-27a-3p mimic* did not inhibit *psiCHECH2-Alp 3'UTR* luciferase activity. Error bars indicate SD. * $p<0.05$.
